# Effect of atmospheric nonthermal plasma on physicochemical, morphology and functional properties of sunn pest (*Eurygaster integriceps*)‐damaged wheat flour

**DOI:** 10.1002/fsn3.2868

**Published:** 2022-04-26

**Authors:** Amir Tavakoli Lahijani, Fakhri Shahidi, Mahmoud Habibian, Arash Koocheki, Behdad Shokrollahi Yancheshmeh

**Affiliations:** ^1^ Department of Food Science and Technology Faculty of Agriculture Ferdowsi University of Mashhad (FUM) Mashhad Iran; ^2^ 113401 Chemistry and Chemical Engineering Research Center of Iran Tehran Iran; ^3^ Food Safety Research Center (salt) Semnan University of Medical Sciences Semnan Iran

**Keywords:** flour modification, gluten, nonthermal plasma, sunn pest‐damaged wheat

## Abstract

To improve the quality of sunn pests (*Eurygaster integriceps*)‐damaged wheat flour, the effects of nonthermal plasma on physicochemical, rheological, functional, and microstructural properties were investigated. Gas type (air and oxygen), voltage (22 and 25 volts), and time (0, 2, 4, 6, 8, and 10 min) were the variables of the experiments conducted using a completely randomized design with three replications. The results show that with increasing voltage and time of plasma treatment, the pH decreased significantly (*p* ≥ .05), and brightness parameter, yellow–blue parameter, water‐solubility, water absorption, oil absorption, and swelling power increased significantly (*p* ≥ .05). The duration of plasma treatment, voltage, and change in input gas from air to oxygen did not significantly change the gluten index, particle size, and negative electric charge of flour particles, and the amount of zeta potential of samples. Differential calorimetric analysis showed the first and second peaks of the thermogram in the range 55–99°C and also 114–99°C. Infrared spectroscopy (FT‐IR) showed hydroxyl group, CH bonds, C=O bonds, as well as the presence of types I and II amide bonds in the structure. Microstructural results indicated that plasma treatment reduced the particle size and increased particle sorting. By Increasing voltage and the duration of plasma treatment, peak viscosity, final viscosity, breakdown viscosity, pasting time and temperature significantly increased and setback viscosity decreased (*p* ≥ .05), which reduced retrogradation which improved the dough stability during the cooling process.

## Graphical abstract

Cold Plasma treatment improved the properties of sunn pest‐damaged wheat flour, including color parameters, water absorption capacity, solubility, oil absorption capacity, swelling strength, pasting time as well as, pasting temperature and peak, final and breakdown viscosity.

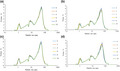

## 1 INTRODUCTION

The quality of wheat flour is an essential technological parameter to produce bread and other cereal products. Gluten is the major protein in wheat which distinguishes this grain from other cereals, plays an important role in the formation of the dough network, and also confers viscosity and elasticity properties to the dough (Day et al., 2006; MacRitchie, 1984). Contrariwise, any factor which negatively affects and destabilizes gluten structure would lead to the significant quality reduction in bread in terms of loaf volume, texture, etc. Sunn Pest (*Eurigaster integriceps*) is the most important destructive insect species for wheat causing pre‐harvest damage and reducing the yield and the baking quality of wheat flour (Armstrong et al., 2019). The insect infests the grains by its saliva which contains proteolytic enzymes that progressively degrade and breakdown the gluten structure and consequently lead to the production of low‐volume bread with coarse texture (Every, 1992; Matsoukas & Morrison, 1990). The presence of 3%–5% damaged wheat seriously affects the rheological and baking quality of flour, and higher levels of damage above 10% would generally be downgraded to be used for feed applications (Hariri et al., 2000; Karababa & Ozan, 1998).

The proposed methods for improving the structural damage caused by sunn pest are mainly based on the separation of damaged and normal wheat or reinforcement of new bonds in damaged gluten structure using chemical oxidizing compounds such as potassium bromate, ascorbic acid, iodine, chlorine dioxide, and benzoyl peroxidase (Patrignani et al., 2014), which would stabilize the protein network by oxidizing thiol group into disulfide bonds and formation of cross‐linking of proteins to restore the strength of the gluten network and consequently improve the bakery properties of damaged wheat (Saeed & Howell, 2004). However, there are several reports of these substances such as potassium bromate and ammonium persulfate being dangerous for the health (Ahmad et al., 2014; Kurokawa et al., 1990). Thermal methods can also be used to inactivate the proteolytic enzymes but it can negatively affect the viscoelastic structure of gluten. There is growing interest in focusing on the development of safe and inexpensive nonthermal technologies and reducing the application of chemical agents in food. Nonthermal plasma (NTP) is a technology that can be used to confer different functional groups on the surface of materials or the effect of its partial oxidation on food compounds and improve the final properties of food matrixes. NTP as the fourth state of the matter refers to an ionized gas containing electrons, atoms, ions, or neutral gas molecules, ultraviolet photons, free radicals, reactive species, and excited molecules and atoms. The free electric charges (electrons and ions) make plasma electrically conductive, internally interactive, and strongly responsive to electromagnetic fields (Fridman et al., 2008). In NTP, typically the electrical discharges with high voltage provide local thermodynamic equilibrium among the species that exists, whereas, in the latter, cooling of ions and uncharged molecules are more effective than energy transfer from electrons, and the gas remains at low temperature; for this reason, none equilibrium plasma is also called NTP or cold plasma (Misra et al., 2016). In recent years, the application of NTP on food has been studied in the field of microbial inactivation efficiency, especially for temperature‐sensitive products, and food packaging as well. Limited researches have shown that NTP induced the structural modification of macromolecules and improve the functional properties of gluten in wheat flour (Bahrami et al., 2016; Menkovska et al., 2014; Misra et al., 2015), starch (Pankaj et al., 2015; Thirumdas et al., 2015; Zhang et al., 2014), and whey protein (Segat et al., 2015). In different types of plasma, it is reported that the formation of reactive species especially oxygen and nitrogen reactive species (ROS and NOS) can proceed with some chemical reactions, such as oxidation, polymerization, and cleavage reactions (Afshari & Hosseini, 2014; Pankaj et al., 2014; Schlüter et al., 2013). On the other hand, Ozone as one of the most effective reactive species in NTP can modify wheat flour functionality (Chittrakorn, 2008; Misra et al., 2015; Sandhu et al., 2012). This study aims to identify the potential of NTP to improve the technofunctional quality of damaged protein to reinforce the gluten network of sunn pest‐damaged wheat flour.

## 2 MATERIALS AND METHODS

### 2.1 Materials

Wheat grains (*Triticum aestivum*) were prepared from the laboratory of the Iran flour factory. Sunn pest‐damaged wheat grains were selected. To produce flour with 10% damaged percentage, 20 g of damaged wheat grains was mixed with 180 g of normal wheat. The wheat grains samples were then milled by a laboratory mill, mesh 21 μm. The flour samples were stored in glass containers in the refrigerator (temperature 4°C).

### 2.2 Plasma treatment of wheat flour

The dielectric barrier discharge (DBD) plasma system consists of two parallel circular plates (*R* = 24 cm) electrodes with a circular (Nik Plasma Teck). Glass plate (4 mm thick) was used as dielectric, which is placed on one of the electrodes. In this study, the treatments included voltage at two levels (22 and 25 kV) with a DC pulse generator (sinusoidal 50 Hz voltage), the contact time of the samples was at levels (0, 2, 4, 6, 8, and 10 min), and the gas types were air and oxygen in atmospheric pressure to produce uniform plasma. For all experiments, a thin layer of flour sample (8 g) was evenly spread over the base area of the ground electrode and directly exposed to the plasma. The electrodes were fixed in the system by two Plexiglas retaining tools and one of the electrodes was linked to movable pistons via fiber to adjust the distance of the electrode and the surface of the sample.

### 2.3 Physicochemical determinations

#### 2.3.1 pH determination

The pH of flour samples was measured using pH meter model 827 (Metrohm, Switzerland).

#### 2.3.2 Gluten index

Gluten index content was measured using the automatic gluten washing machine (model 2200, Brabender). The total weight of the remaining gluten on the sieve is defined as the gluten index (AACC‐38‐12, 11‐38, and 12‐38).

#### 2.3.3 Color parameters

A digital colorimeter (CR‐410 model, Konita Minolta Sensing, Japan) was used to determine the color parameters (L*, a*, and b*) of flour samples. A plastic plate with a diameter of 58 mm and a depth of 15 mm was used to place the sample on it and measure the color parameters. The initial calibration of the device was performed using standard white tiles (Sandhu & Kochhar, 2012).

### 2.4 Solubility of flour

Flour solubility was assessed based on the Ravaghi et al.’s (2010) method, with some modifications. The sample (4 g) in 60 ml of distilled water was homogenized by Ultratorax for 10 min at 10,000 rpm and then centrifuged at 3000 *g* for 10 min. The solubility of flour or the total amount of solids dissolved in the supernatant was determined by dividing the weight of dried supernatant by the amount of sample (Ravaghi et al., 2010).

### 2.5 Water and oil absorption capacity

Measurement of water and oil absorption capacity was performed based on the method of Feyzi et al. (2015). One gram of flour was poured into a centrifuge tube. Then 10 ml of sunflower oil or distilled water was added to the flour and mixed for 2 min using Vertex (Reax Control model, Heidolph). Then, the samples were placed at room temperature for 30 min and they were centrifuged at 3000 *g* for 20 min and the supernatant (oil or water) was separated. Water and oil absorption capacity was calculated as the volume of water or oil absorbed (ml) per gram of flour through the following equation:

(1) $$\begin{array}{cc} \text{WAC}\;\text{or}\;\text{OAC} & =\text{Flour}\;\text{Weight}/(\text{Initial}\;\text{Volume}\;\text{of}\;\text{Water}\;\text{or}\;\text{Oil}\\ & \;‐\text{Volume}\;\text{of}\;\text{Water}\;\text{or}\;\text{Oil}\;\text{Separated}\;\text{after}\;\text{Centrifugation}) \end{array}$$

### 2.6 Swelling Index

The measurement of swelling strength of flour samples was done based on the method of Obadi et al. (2018), with some changes. In this method, 0.5 g of flour was poured into test tubes with lids. Then, 15 g of distilled water was added to the flour, and after stirring, it was heated in a hot water bath at 90°C for 30 min. The heated samples were rapidly cooled to room temperature with a cold water bath and then centrifuged at 3000 × 3 *g* for 10 min and the supernatant was separated. Then, the weight of the pipe along with the sediment was carefully measured and after reducing the empty weight of the pipe, the weight of the remaining phase (sediment) was calculated. The swelling index is determined using the following equation as the percentage of residual sediment.

(2) SwellingIndex=(FlourWeight/SedimentPhaseWeight)

### 2.7 Particle size and zeta potential

Particle size distribution and zeta potential (ζ) were measured using a Zetasizer Nano ZS Device (Zen 3600, United Kingdom). From each sample, the solution (concentration of 1 mg/ml) (distilled water solvent) was prepared and passed through a 1 μm filter before measurement (Chen et al., 2019).

### 2.8 Differential scanning calorimetry

The flour (10 mg) was weighed in an aluminum pan and heated to 20 to 130°C with a heating rate of 10°C/min. Thermos‐analytical parameters including glass transfer temperature, denaturation temperature, number of endothermic peaks, and enthalpy of denaturation were measured (Caballero et al., 2004).

### 2.9 Fourier Transfer Infrared Analysis (FTIR)

Fourier transform infrared (FT‐IR) spectroscopy was performed to identify the compounds and investigate the secondary structure of gluten protein according to Misra et al. (2015), using Bruker IFS (66, Germany) with Specac Golden Gate diamond ATR accessories. All of these spectroscopies were performed between wavelengths of 400 to 4000 cm^−1^.

### 2.10 Scanning Electron Microscopy (SEM)

Microstructural studies of the samples were performed using Scanning Electron Microscopy (model TESCAN3VEGA, ) with magnification ranging 250, 1000×, 3000×, and 5000× (Sidhu et al., 1990). The surfaces of the samples were first covered with a layer of gold. Then, the samples were glued to the metal holder of the device and guided into the system. In the test chamber, the samples were rotated by a mechanical arm for viewing and magnification.

### 2.11 Rapid Visco Analysis (RVA)

The pasting and rheological characteristics ability of flour solutions (3.5 g of sample based on the dry weight in 25 g of distilled water) was investigated by the rapid viscosity analyzer (RVA) model (Starch Master 2, Pattern Instruments) as a function of temperature.

### 2.12 Statistical analysis

All experiments were performed in three replications. Statistical analysis of the data was performed using a completely randomized design and comparison of means using Duncan's test at a significance level of *p* < .05. The results were analyzed using SPSS software (version 16) and Excel 2007 software was used to draw the graphs. Statistical analysis of data was performed using Microsoft Excel.

## 3 RESULTS AND DISCUSSION

### 3.1 Physicochemical characteristic

#### 3.1.1 pH

The results of pH were presented in Table 1. With increase in the duration of plasma treatment, the pH of the samples decreased significantly (*p* < .05). The highest pH was related to the control sample with a rate of 6.44 and the lowest pH in the plasma‐treated flour sample for 10 min reached 5.89. Misra et al. (2015) stated that during plasma treatment, generated ozone gas, UV waves, active species produced from oxygen and nitrogen, as well as electrons have strong oxidizing properties or the ability to modify the structure of macromolecules including changes in the functional groups of amino acids which will eventually lower the pH in this complex system. It is mainly influenced by the reaction with reactive oxygen species that leads to increased formation of intramolecular disulfide bonds, as well as the acid sulfonic or sulfuric formation. Reactive nitrogen species form nitric acid (HNO_2_) and nitric acid (HNO_3_) derived from NO and NO_2_ that eventually lower the pH (Segat et al., 2015) as well as the reaction of ozone molecules which oxidize the sulfhydryl group. Banura et al. (2018) and Thirumdas et al. (2017) reported that plasma causes the formation of acidic groups such as carbonyl, carboxyl, and peroxides in starch samples which leads to lower pH.

**TABLE 1:** Physicochemical and color parameters of plasma‐treated flour

Gas type	Voltage	Time	pH	Gluten index	L*	a*	b*
Air	22	0	6.30 ± 0.01^a^	55.70–±1.53^b^	83.20 ± 0.06^h^	−1.19 ± 0.03^a^	18.92 ± 0.02^cd^
		2	6.25 ± 0.02^b^	55.15–±1.57^e^	83.20 ± 0.05^h^	−1.26 ± 0.01^b^	18.92 ± 0.02^cd^
		4	6.17 ± 0.01^c^	54.66–±0.76^g^	83.21 ± 0.06^h^	−1.22 ± 0.03^ab^	18.91 ± 0.01^d^
		6	6.08 ± 0.01^e^	55.22–±0.68^de^	83.25 ± 0.08^g^	−1.25 ± 0.02^b^	18.94 ± 0.03^cd^
		8	6.03 ± 0.01^g^	55.50–±0.55^c^	83.37 ± 0.06^g^	−1.27 ± 0.01^b^	18.94 ± 0.01^c^
		10	6.15 ± 0.01^cd^	55.03–±0.56^f^	83.72 ± 0.06^e^	−1.29 ± 0.04^b^	18.95 ± 0.02^bc^
	25	0	6.30 ± 0.01^a^	55.70–±1.53^b^	83.20 ± 0.06^h^	−1.19 ± 0.03^a^	18.92 ± 0.02^cd^
		2	6.01 ± 0.01^h^	55.13–±0.37^e^	83.26 ± 0.01^a^	−1.30 ± 0.05^bc^	18.90 ± 0.05^d^
		4	5.92 ± 0.01^j^	55.43–±0.96^c^	83.62 ± 0.05^f^	−1.30 ± 0.03^c^	18.92 ± 0.01^cd^
		6	5.94 ± 0.01^i^	55.80–±0.51^b^	83.97 ± 0.05^d^	−1.39 ± 0.04^d^	18.94 ± 0.01^cd^
		8	5.89 ± 0.01^K^	56.20–±0.48^a^	84.10 ± 0.06^c^	−1.43 ± 0.02^d^	18.94 ± 0.02^cd^
		10	5.88 ± 0.01^k^	55.68–±0.47^b^	84.26 ± 0.02^a^	−1.44 ± 0.01^d^	18.95 ± 0.03^bc^
Oxygen	22	0	6.30 ± 0.01^a^	55.70–±1.53^a^	83.20 ± 0.06^h^	−1.19 ± 0.03^a^	18.95 ± 0.02^cd^
		2	6.23 ± 0.02^b^	54.53–±0.80^g^	83.27 ± 0.04^hi^	−1.21 ± 0.06^ab^	18.90 ± 0.05^d^
		4	6.13 ± 0.01^d^	55.41–±1.37^c^	83.61 ± 0.08^f^	−1.26 ± 0.01^b^	18.92 ± 0.01^cd^
		6	6.02 ± 0.02^gh^	55.05–±0.83^f^	84.08 ± 0.06^cd^	−1.23 ± 0.02^ab^	18.94 ± 0.01^cd^
		8	5.94 ± 0.01^i^	55.15–±0.32^e^	84.19 ± 0.01^b^	−1.29 ± 0.04^bc^	18.94 ± 0.02^cd^
		10	5.94 ± 0.01^i^	55.11–±0.25^e^	84.30 ± 0.04^a^	−1.31 ± 0.03^bc^	18.95 ± 0.03^bc^
	25	0	6.30 ± 0.01^a^	55.70–±1.53^b^	83.20 ± 0.06^h^	−1.19–±0.03^a^	18.92 ± 0.02^cd^
		2	6.02 ± 0.02^gh^	54.55–±0.37^g^	83.27 ± 0.04^hi^	−1.32–±0.04^c^	18.97 ± 0.01^ab^
		4	6.05 ± 0.02^f^	54.80–±0.38^a^	83.61 ± 0.08^f^	−1.31–±0.03^a^	19.00 ± 0.02^a^
		6	5.94 ± 0.01^i^	55.61–±0.30^bc^	84.08 ± 0.06^cd^	−1.35–±0.04^cd^	18.98 ± 0.01^ab^
		8	5.89 ± 0.01^k^	55.31–±0.60^d^	84.19 ± 0.01^b^	−1.40–±0.03^d^	18.99 ± 0.02^a^
		10	5.91 ± 0.01^j^	55.45–±1.53^c^	84.30 ± 0.04^a^	−1.45–±0.04^d^	19.00 ± 0.02^a^

#### 3.1.2 Gluten Index

The gluten index indicates the ratio of strong gluten to weak gluten and in fact, indicates the quality of gluten. As shown in Table 1, plasma treatment duration significantly (*p* < .05) increased the gluten index. The highest amount of gluten index (68.3%) was for the treatment time of 8 min and the lowest amount was for the control sample (53.6%). Pankaj et al. (2018) suggested that oxidative changes in proteins may alter their molecular weight and solubility which consequently alter their ability to form a gluten network. It seems that oxidation of proteins by cold plasma is a major phenomenon that can improve protein solubility and technological properties of flour mainly because of an increase in the number of formation and strength of new disulfide bonds (Misra et al., 2015). High levels of cold plasma energy alter protein and lipids; however, increasing the oxidation time or high oxidant concentration can reduce the gluten index by degrading high molecular weight fractions of gluten (Sheikholeslami et al., 2009).

#### 3.1.3 Color parameters

The color of wheat flour is one of the important characteristics that consequently impact the color of the final products and their visual acceptance. Table 1 presents the color parameters index including L * (brightness), a * (red‐green), and b * (yellow‐blue) at different times of plasma treatment. As can be seen with increasing the duration of plasma treatment, the brightness and b * of flour samples increased significantly (*p* < .05); however, the index a * (*p* < .05) was significantly decreased. The largest amount of brightness index, L*, was related to the sample treated with plasma for 10 min and voltage of 25 and the lowest amount of brightness was observed in the control sample. Regarding a *, the highest value was related to the control sample and the lowest value of the index was for the sample treated with plasma for 10 min. For b * index, the largest value was in the sample treated for 10 min and the lowest value of b* index was in the sample treated within 2 min. Pal et al. (2016) and Thirumdas et al. (2015) demonstrated an increase in whiteness with increasing the L * index. The researchers attributed the phenomenon to the fact that atmospheric plasma contains large amounts of active molecules, such as ozone, nitric oxide, peroxide, and hydroxyl radicals, which are produced with minimal energy at room temperature for a few seconds to a few minutes. They react strongly with the color compounds in the flour, which causes oxidation in them, which makes the color lighter in the final product, increasing the L * parameter and decreasing the a * and b * at the same time. These results indicate that cold plasma can oxidize the pigments of wheat flour such as carotenoids, lutein, that have double conjugated bonds. Similar results about the effect of ozone on wheat flour samples were reported by Sui et al. (2016) that a bright white appearance for the samples was obtained by decomposing the existing natural yellow pigments. According to the results of this study, plasma can be used as a potential alternative to the chemical bleaching agent in wheat flour.

### 3.2 Solubility index of flour

Table 2 shows the solubility index of plasma‐treated flour samples in water in different voltages, plasma treatment time, and input gases. Statistical analysis of the results showed that the voltage and treatment time had a significant effect on the water‐solubility parameter and the increase in voltage and time caused an increase in the water solubility (*p* < .05). Altering gas type from air to oxygen significantly reduced the solubility parameter in water (*p* < .05). The highest solubility was obtained in the 25 kV‐air‐10 m treatment at 9.11% and the lowest in the control treatment at 8.73%. The results of this study were following the findings of Obadi et al. (2018), as well as Lee et al. (2017), who in a separate study examined the effect of ozone on physicochemical and functional properties of wheat flours, which was caused by starch oxidation changing the structure of starch and breaking down its chains. Various researchers have also stated in their studies that the application of cold plasma can well reduce the hydrophobic properties of natural polymers and improve their functional properties such as solubility because of free radicals, active species, and breaking the superficial molecular layer in macromolecules, especially protein and starch and subsequently creating functional groups at the polymer surface by the interaction of these functional groups with gas molecules (Bußler et al., 2015; Misra et al., 2015; Segat et al., 2014). Sarangapani et al. (2015) reported that the water‐solubility parameter significantly increased by increasing the plasma treatment time up to 10 min and increasing the power to 50 w because of the breakdown of amylose and amylopectin chains in starch resulting in the formation of lower molecular weight components which increases the solubility of the samples.

**TABLE 2:** Functional parameters of plasma‐treated flour samples

Gas type	Voltage	Time	Solubility index %	Water absorption capacity %	Oil absorption capacity %	Swelling index (ml)
Air	22	0	8.73 ± 0.01^h^	150.41 ± 2.00^gh^	161.61 ± 0.41^f^	14.25 ± 0.15^h^
		2	8.75 ± 0.01^gh^	151.74 ± 1.24^gh^	162.57 ± 0.42^ef^	14.50 ± 0.00^g^
		4	8.77 ± 0.01^g^	153.59 ± 1.92^ef^	162.66 ± 0.53^ef^	14.76 ± 0.05^f^
		6	8.81 ± 0.01^f^	152.78 ± 1.16^fg^	162.64 ± 0.37^ef^	14.96 ± 0.05^e^
		8	8.86 ± 0.01^e^	153.41 ± 1.34^ef^	162.71 ± 0.48^ef^	15.00 ± 0.00^d^
		10	8.87 ± 0.01^e^	154.16 ± 0.70^de^	162.80 ± 0.15^ef^	14.93 ± 0.11^e^
	25	0	8.73 ± 0.01^h^	150.41 ± 2.00^gh^	161.61 ± 0.41^f^	14.25 ± 0.15^h^
		2	8.94 ± 0.01^d^	153.67 ± 1.73^ef^	164.03 ± 0.21^cd^	14.71 ± 0.02^f^
		4	9.03 ± 0.01^b^	154.99 ± 1.06^de^	164.63 ± 0.53^bc^	14.88 ± 0.07^ef^
		6	9.04 ± 0.01^b^	156.55 ± 0.80^bc^	165.07 ± 0.29^ab^	15.25 ± 0.05^c^
		8	9.09 ± 0.01^a^	157.77 ± 0.60^ab^	165.04 ± 0.20^ab^	15.40 ± 0.05^b^
		10	9.11 ± 0.01^a^	158.31 ± 0.66^a^	165.85 ± 0.41^a^	15.61 ± 0.05^a^
Oxygen	22	0	8.73 ± 0.01^h^	150.41 ± 2.00^gh^	161.61 ± 0.41^f^	14.25 ± 0.15^h^
		2	8.75 ± 0.01^gh^	151.03 ± 2.04^gh^	162.55 ± 0.72^ef^	14.46 ± 0.07^g^
		4	8.75 ± 0.01^gh^	152.14 ± 1.36^gh^	162.69 ± 0.10^ef^	14.73 ± 0.07^f^
		6	8.80 ± 0.01^f^	152.64 ± 0.23^fg^	162.70 ± 1.05^ef^	14.76 ± 0.05^f^
		8	8.82 ± 0.02^f^	153.33 ± 0.72^ef^	162.71 ± 0.31^ef^	14.86 ± 0.11^e^
		10	8.85 ± 0.01^e^	154.22 ± 1.16^de^	162.79 ± 0.38^ef^	14.93 ± 0.11^e^
	25	0	8.73 ± 0.01^h^	150.41 ± 2.00^gh^	161.61 ± 0.41^f^	14.25 ± 0.15^h^
		2	8.92 ± 0.02^d^	152.54 ± 1.03^fg^	163.49 ± 1.16^de^	14.73 ± 0.11^f^
		4	8.96 ± 0.01^ef^	154.42 ± 0.56^de^	164.02 ± 0.39^cd^	14.90 ± 0.10^e^
		6	8.97 ± 0.00^c^	155.94 ± 0.54^cd^	164.51 ± 0.35^bc^	15.03 ± 0.05^d^
		8	8.99 ± 0.01^c^	156.32 ± 0.13^bc^	164.62 ± 0.19^ab^	15.21 ± 0.07^c^
		10	8.99 ± 0.01^a^	156.61 ± 0.71^bc^	164.99 ± 0.17^ab^	15.25 ± 0.02^c^

### 3.3 Water and oil absorption capacity

Water absorption capacity is one of the important factors in the quality of wheat flour, depending on the quantity and quality of protein, starch, and its molecular weight, as well as the amount of fiber. Increasing water absorption causes the gluten network and finally the dough to form a suitable and uniform texture for bread production, which in turn improves the dough's ability to flatten, increases the volume of the bread, and increases the shelf life of the product. As shown in Table 2, the amount of water absorption capacity of wheat flour samples was significantly increased by the increase in voltage and duration of plasma treatment (*p* < .05). Altering gas type from air to oxygen significantly reduced the water absorption capacity (*p* < .05). According to the results, the highest amount of water absorption was related to a 25 kV‐air‐10 m treatment of 158.31% and the lowest amount of water absorption was in the control treatment of 150.41%. Obadi et al. (2018) and Lee et al. (2017) reported the increasing trend of the water absorption capacity of wheat flour during ozone treatment. Ozone is the major active atmosphere in air cold plasma that has an oxidizing effect. Opening of protein chains, the placement of more hydrophilic groups on the surface, as well as the breakdown of long amyloid chains, and the formation of lower molecular weight components increase the water absorption of macromolecules (Segat et al., 2014; Thirumdas et al., 2015). The surface oxidation of granules occurs in contact with generated reactive species and increases with the change in surface charge, and the placement of polar functional groups on the surface of the granule leads to absorbing more water. Protein depolymerization increases the surface‐to‐volume ratio of the polymer particles and increases their reactivity with water molecules (Bußler et al., 2015). The presence of sugars such as sucrose, fructose, and glucose increases viscosity and water absorption capacity. Retaining more water in the structure. The formation of active groups increases the surface activity and hydrophilicity of starch granules (Banura et al., 2018).

Oil absorption capacity is more of a physical phenomenon as the compounds and biopolymers in the sample cause oil droplets to be trapped inside. Many researchers attribute oil uptake to nonpolar protein chains that may bind to the oil's hydrocarbon side chains. The higher the oil uptake capacity, the higher the amount of nonpolar amino acids in the side chain (Bußler et al., 2015). In addition to the amino acid content of the wheat flour, the protein structure as well as the surface polarity, are effective on oil absorption capacity. The majority of the changes in oil absorption capacity have been attributed to changes in proteins during plasma treatment showing the surface and structural modification of protein and fiber matrix in comparison to starch molecules (Misra et al., 2015). Voltage and treatment time of cold plasma treatment caused a significant increase in oil absorption of wheat flour samples, but altering gas type from air to oxygen significantly reduced the oil absorption capacity (*p* < .05). According to Table 2, the highest amount of oil uptake was for in the treated sample of 25 kV‐air‐10 m treatment at 165.85% and the lowest amount in the control sample at 161.11%. This indicates the exposure of nonpolar groups in protein as well as increasing in surface hydrophobicity of flour particles which matches the results of Bußler et al. (2015) stated that cold plasma increased the water and oil absorption capacity depending on the plasma treatment time and protein composition.

### 3.4 Swelling Index

The swelling index indicates the rate of reactions between starch chains in amorphous and crystalline regions (Chan et al., 2009). The increase in swelling strength is influenced by the ratio of amylose to amylopectin, the size of the molecules, the length of the amylopectin side chain, and the spatial shape of the molecule (Singh & Kaur, 2004). According to the results obtained from Table 2, voltage and treatment time of plasma treatment significantly increased the swelling index of flour samples (*p* < .05) but altering gas type from air to oxygen significantly reduced the oil absorption capacity (*p* < .05). The highest amount of swelling was related to the sample treated at 25 kV‐air‐10 m at 15.61 g and the lowest amount of swelling strength was obtained in the control treatment at the rate of 14.25 g. Increased swelling power is directly related to water absorption and solubility of flour particles due to cold plasma treatment. In other words, to create the functional properties of flour particles, these particles must first have a good solubility in water, which will lead to higher water absorption (Sarangapani et al., 2015). The presence of hydrophilic groups, the formation of new hydrogen bonds, as well as the electric charge density at the surface of the starch granules plays a vital role in this process. Decreasing the pH of cold plasma‐treated flour samples makes the carboxyl groups in starch particles less inclined to lose protons, and this event creates more hydrogen bonds in flour samples during dissolution in water, so weak forces or bonds, such as van der waals bonds, that are excited in this case would be increased and the physical binding of water to the flour molecules and, ultimately, the swelling power will be higher (Thirumdas et al., 2015). On the other hand, according to Pankaj et al. (2018), cold plasma treatment can be used as an effective method to improve the functional properties of starch causing acetylation of starch which increases the hydrophilic properties of starch and improves the swelling strength.

### 3.5 Particle size and zeta potential

**FIGURE 1:**
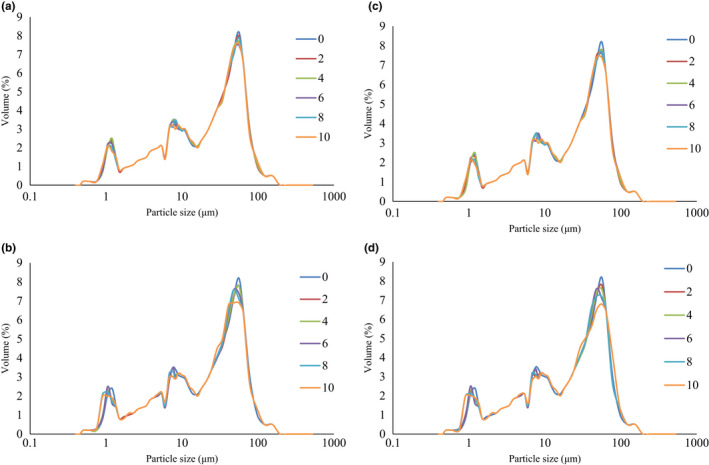
Particle size distribution of plasma‐treated samples (a): Oxygen, at 22 kv, (b) Oxygen, at 25 kv, (c) Air, at 22 kv, and (d) Air, at 25 kv at different times

The results of the particle size distribution of treated wheat flour are shown in Figure 1a–d. Three obvious peaks were yielded. The first peak is in the range 1–1.5 micrometers, the second peak is in the range 7–8 μm, and the third peak is in the range 55–56 μm. Also, in terms of the ratio of the particles, the majority of the particles are in the range of the third peak and then observed in the second and first peak, respectively. Therefore, more than 50% of the cold plasma‐treated flour particles have a size in the range of 10–100 μm. The results showed that the time and intensity of the treatment and the change in gas type from air to oxygen did not change the particle size of the flour samples.

Table 2 indicates the zeta potential evaluation of plasma‐treated flours at different voltages, time, and gas types. The zeta potential of the samples ranged from −22.58 to −21.52 mV. No significant change on the negative bar of flour particles was observed with increasing the duration and intensity of cold plasma treatment as well as gas type. The higher the zeta potential, results in higher electrostatic, force between molecules, which prevents the accumulation of particles. This will increase the stability of the product and its resistance to sediment. Increasing the repulsion force between paricles can lead to create more stable systems. However, sometimes a completely different goal is pursued, and by removing or reducing repulsive forces, the formation of large masses is accelerated and smooth operation becomes easier. According to Miura et al. (2011), by changing the balance between repulsive forces and gravity between particles (e.g., by applying plasma flow), the viscosity of the solution can be changed and adjusted.

### 3.6 Differential scanning calorimetry

According to the results in Table 3, G1 and G2 indicate the first and second temperature peaks, ΔH_1_ and ΔH_2_ indicate the enthalpy of the reaction at the first and second peaks, respectively. The results of evaluations showed that the first enthalpy in all treatments is higher than the second one. On the other hand, with increasing the duration of cold plasma treatment at 22 kV, the first and second enthalpies decreased. The amount of ΔH_1_ increased from 26.32 j/g in the control sample to 24.03 j/g in the treatment of 22 kV‐air‐10 m. This is while at 25 kV, the first and second enthalpy values increased so that the amount of ΔH_1_ increased from 28.24 j/g in 25 kV‐oxygen‐2m treatment to 31.66 j/g in 25 kV‐Air‐10 m. Also, the change in voltage from 22 to 25 kV increased the enthalpy at both peaks, while the change in gas type from air to oxygen did not make much difference between the flour treatments. In the thermal evaluation of flour samples, the presence of two thermal peaks in the range 55–99°C and also 114–99°C has been observed that due to the convexity of these peaks, the total reaction energy of these peaks was negative, or more accurately the exothermic reaction. Zaidul et al. (2008) evaluating the thermal characteristic of a mixture of wheat flour and potato starch pointed out that the first peak with a positive enthalpy occurred in the temperature range 25–45°C, which indicates the enthalpy of starch gelatinization in the presence of residual moisture in the flour. The researchers reported a high enthalpy at this stage, at 3.5 j/g, which is much lower than the first peak enthalpy in the present study and also the presence of a weak peak with a positive enthalpy in the range of 80°C due to the breakdown of hydrogen bonds between the amylose and amylopectin chains, which led to the release of energy. Similar findings are stated in a study by Eliasson (1983), on the thermal evaluation of wheat flour reporting a long peak in the 60°C temperature range with a negative enthalpy. Also, the second convex peak was in the temperature range 87°C, which was smaller and had a negative enthalpy (exothermic) of 1.48 cal/g attributing the second peak to the decrease in moisture content in this temperature range in starch–gluten systems. Particularly, by reducing the moisture available to the starch at higher temperatures, the second peak rises to higher temperatures, and the power of gluten in water absorption will cause a peak in this range. In the present study, with the increase in cold plasma treatment, the rate of moisture exit due to starch depolymerization is higher and this causes the second peak to rise to higher temperatures. However, due to the failure of hydrogen bonds in the amylose chains, the peak enthalpy decreases.

**TABLE 3:** Zeta potential of plasma‐treated flour samples

Gas type	Voltage	Time	Zeta potential (mV)	Zeta deviation
Air	22	0	−21.52^a^	7.50
		2	−21.87^a^	7.53
		4	−21.84^a^	7.51
		6	−21.97^a^	7.55
		8	−22.01^a^	7.41
		10	−22.03^a^	7.50
	25	0	−21.52^a^	7.50
		2	−22.36^a^	7.38
		4	−22.44^a^	7.49
		6	−22.49^a^	7.40
		8	−22.51^a^	7.43
		10	−22.58^a^	7.44
Oxygen	22	0	−21.52^a^	7.50
		2	−22.07^a^	7.44
		4	−22.11^a^	7.48
		6	−22.15^a^	7.65
		8	−22.16^a^	7.54
		10	−22.16^a^	7.41
	25	0	−21.52^a^	7.50
		2	−22.25^a^	7.40
		4	−22.23^a^	7.38
		6	−22.35^a^	7.42
		8	−22.36^a^	7.40
		10	−22.39^a^	7.56

### 3.7 Fourier Transfer Infrared Analysis (FTIR)

**FIGURE 2:**
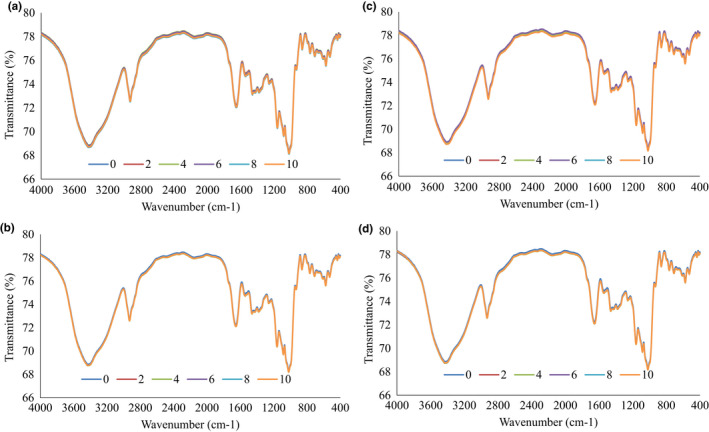
FTIR spectrum of flour plasma treatments (a): Oxygen, 22 kv, (b) Oxygen, 25 kv, (c) Air, 22 kv, and (d) Air, 25 kv at different times

As can be deduced from Figure 2a–d, several wavelengths have peaks including 3430.42, 2929.46, 1653.52, 1458.35, 1159.16, 1083.51, 1019.55, 860.52, 528.32, and 575.25 per cm. These points represent special functional groups in wheat flour. On the other hand, there is not much difference between the FTIR spectrum of flour treatments. Thirumdas et al. (2017) also reported that plasma did not affect the FTIR pattern of rice starch samples. Kamara et al. (2009) attributed the peak in the range of 3423 cm^−1^ to the hydroxyl groups, also a peak in the range of 2926 cm^−1^ referred to C‐H bonds. The wavenumber 1643 cm^−1^ also indicates the presence of amide bonds of the first type in the structure of flour. In our study, a peak in the wavenumber of 1458 cm^−1^ has also been observed, which probably indicates the second type of amide bonds in the flour structure. According to Amir et al. (2013), the presence of a peak in the range 500–650 cm^−1^ was related to the bending of the C‐H group in the aromatic groups or the late bending related to the C‐OH bond.

### 3.8 Scanning electron microscopy (SEM)

**FIGURE 3:**
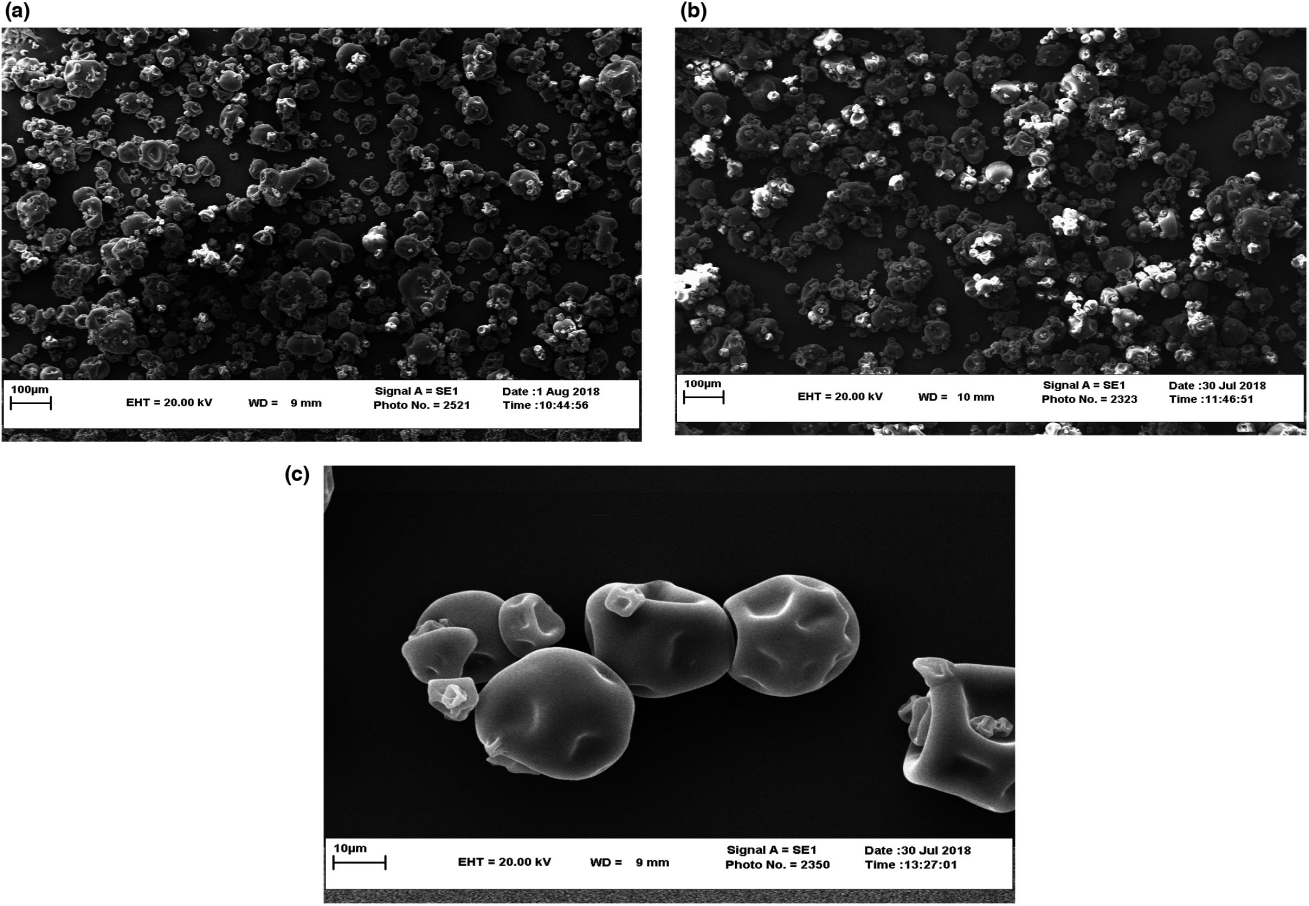
Scanning electron micrographs (SEM) of (a) control sample at 250 magnifications, (b) plasma treated flour sample 25 kv‐Oxygen‐4 min at 250 magnifications; and (c) plasma treated flour sample 25 kv‐Air‐4 min at 3000 magnifications

There were no significant differences in the electron micrographs performed at 22 kV, so these treatments were excluded. Based on Figure 3a obtained from the control sample, at 250 magnifications, particles with various dimensions can be seen. According to the 100‐micron index in the image, most of the particles are smaller than this amount. By increasing the voltage to 25 kV, the particles showed a homogeneous structure and more uniformity. In Figure 3b related to 25 kV‐oxygen‐4‐min treatment, the amount of particles with dimensions less than 100 μm appears to be much higher than the control treatment. Figure 3c also shows an image with a magnification of 3000 times of 25 kV‐air‐4 min treatment, which indicates the presence of particles with dimensions of about 20 μm (two times the 10‐μm index in the image). Other treatments with a voltage of 25 kV have also been observed. The results obtained from scanning electron microscopy show that the control sample has an irregular granulation structure compared to other treatments affected by cold plasma treatment and the particles are multifaceted because of the particle size dispersion and very large particles can be seen in it while particle sorting is much higher in plasma‐treated flours (especially at 25 kV). In other words, particles with the same size in flour treatments at 25 kV are more than other treatments, and high size particles that are mostly observed in the control sample are not observed in these treatments.

For example, in a control sample at 250 magnifications, particles of various dimensions and even particles larger than 100 μm can be seen, but this trend cannot be observed in 25 volts kV treatments. This indicates a breakdown in the flour particles and a reduction in particle size. The results of these findings are similar to the research conducted by Thirumdas et al. (2017) in the field of functional and physicochemical properties of cold plasma‐treated rice starch. The researchers noted that the starch granules were irregular, hexagonal, or polyhedral before treatment and that the number of cavities in them was less noticeable. However, by performing the plasma treatment for 10 min at a power of 60 w, cavities were observed on the surface of the granules, which is similar to the results obtained in the 25 kV flour treatments as shown in Figure 3c. The researchers attributed the cavities on the surface of the granules to the penetration of ions and radical groups created when plasma flows into the granules. In another study by Banura et al. (2018), similar results were reported for corn and tapioca starch, stating that cavities were observed on the surface of the granules by the plasma process.

### 3.9 Rapid Visco Analysis (RVA)

Table 4 shows the viscosity parameters of flour‐treated samples. An increase in voltage and treatment time caused a significant increase in peak viscosity of the samples. The pasting properties have a significant effect on the behavior of starch during and after baking. The pasting properties depend more on the structural properties of amylopectin than on amylose (Juliano et al., 1987). Peak viscosity is the highest sample viscosity when starch is gelatinized in water. With increasing temperature and water availability, the crystal structure of the granules disintegrates and amylose and amylopectin are released which leads to an increase in the viscosity. Donelson et al. (2000) reported that the peak viscosity of wheat flour increased with the chlorination process of wheat flour. Varriano‐Marston (1985) reported that oxidation of starch breaks branches on the surface of crystals and creates amorphous parts. These changes increase the porosity of the surface and restore the structure of the starch, allowing water and oil to bind to the surface. Hoseney (1994) reported that chemical modification by oxidation increases the swelling capacity of starch and increases viscosity. Banura et al. (2018) reported that increasing the intensity and time of plasma treatment increased the peak viscosity of the samples.

**TABLE 4:** Results of differential scanning calorimetry (DSC) of plasma‐treated flour samples

Gas type	Voltage	Time	G1 (℃)	ΔH1 (J g^−1^)	G2 (℃)	ΔH2 (J g^−1^)
Air	22	0	58.20^d^	26.32^j^	99.87^h^	7.42^l^
		2	55.67^e^	29.15^f^	98.80^j^	8.51^h^
		4	55.01^e^	29.21^e^	97.94^k^	8.33^j^
		6	55.60^e^	24.90^k^	96.22^l^	7.00^n^
		8	55.83^e^	26.35^j^	96.22^l^	7.30^m^
		10	55.54^e^	24.03^m^	94.87^n^	6.29^q^
	25	0	58.20^d^	26.32^j^	99.87^h^	7.42^l^
		2	62.14^bc^	28.20^h^	112.55^g^	8.73^g^
		4	63.78^abc^	30.09^d^	113.59^b^	9.67^d^
		6	63.67^a^	31.21^c^	113.41^de^	9.79^c^
		8	63.90^ab^	31.59^b^	113.55^b^	10.36^a^
		10	63.58^abc^	31.66^a^	113.34^f^	10.30^b^
Oxygen	22	0	58.20^d^	26.32^j^	99.87^h^	7.42^l^
		2	55.68^e^	26.45^i^	98.80^j^	7.49^k^
		4	55.60^e^	29.11^f^	98.77^j^	8.44^i^
		6	55.51^e^	24.69^l^	97.91^k^	6.88^p^
		8	55.78^e^	24.95^k^	96.02^m^	6.94^o^
		10	55.66^e^	23.86^n^	94.92^n^	6.24q
	25	0	58.20^d^	26.32^j^	99.87^h^	7.42^l^
		2	62.11^e^	28.24^h^	112.52^g^	8.47^g^
		4	63.54^abc^	29.15^f^	113.71^a^	8.95^f^
		6	63.66^abc^	30.19^c^	113.37^ef^	9.76^c^
		8	63.97^a^	29.13^f^	113.49^c^	9.10^e^
		10	63.95^a^	29.02^g^	113.44^cd^	9.13^e^

According to the results in Table 5, oxygen plasma‐treated samples had higher pasting time. The results showed that the increase in voltage and time caused a significant increase in the pasting time of the samples. The pasting time refers to the time when the strands of amylose and amylopectin leave the granules and the viscosity begins to increase. The production of acidic compounds during the process increases the number of bonds between the chains and ultimately increases the viscosity. Delays in viscosity and pasting time are probably due to the initial spatial barrier created.

**TABLE 5:** Viscosity parameters of plasma‐treated flour samples

Gas type	Voltage	Time	Peak viscosity (Cp)	Pasting time (s)	Pasting temperature	Hold viscosity (Cp)	Final viscosity (Cp)	Breakdown (Cp)	Setback (Cp)
Air	22	0	1898.50 ± 3.54^o^	62.25 ± 0.35^l^	67.93 ± 0.10^a^	1017.50 ± 2.12^l^	2118.50 ± 2.12^q^	1548.00 ± 1.41^b^	949.50 ± 2.12^q^
		2	1921.50 ± 3.54^n^	62.25 ± 0.35^l^	50.43 ± 0.10^j^	952.50 ± 2.12^n^	2266.50 ± 2.12^p^	969.00 ± 1.41^n^	1318.50 ± 2.12^n^
		4	2033.50 ± 3.54^l^	77.50 ± 0.71^i^	52.69 ± 0.45^h^	1048.50 ± 2.12^j^	2520.50 ± 2.12^l^	985.00 ± 1.41^l^	1476.50 ± 2.12^j^
		6	2044.50 ± 3.54^k^	64.50 ± 0.71^k^	50.41 ± 0.13^j^	998.50 ± 2.12^m^	2485.50 ± 2.12^m^	1046.00 ± 1.41^f^	1491.50 ± 2.12^h^
		8	2099.50 ± 3.54^i^	148.50 ± 0.71^a^	67.72 ± 0.40^ab^	1121.50 ± 2.12^d^	2561.50 ± 2.12^g^	978.00 ± 1.41^m^	1594.50 ± 2.12^c^
		10	2129.50 ± 3.54^g^	98.50 ± 0.71^g^	56.78 ± 0.31^f^	1142.50 ± 2.12^c^	2588.50 ± 2.12^f^	987.00 ± 1.41^l^	1544.50 ± 2.12^f^
	25	0	1898.50 ± 3.54^o^	62.25 ± 0.35^l^	67.93 ± 0.10^a^	1017.50 ± 2.12^l^	2118.50 ± 2.12^q^	1548.00 ± 1.41^b^	949.50 ± 2.12^q^
		2	2091.50 ± 3.54^j^	146.50 ± 0.71^b^	67.32 ± 0.25^b^	1048.50 ± 2.12^j^	2527.50 ± 2.12^k^	1043.00 ± 1.41^g^	1483.50 ± 2.12^i^
		4	2113.50 ± 3.54^h^	146.50 ± 0.71^b^	67.35 ± 0.22^ab^	1066.50 ± 2.12^h^	2562.50 ± 2.12^g^	1047.00 ± 1.41^f^	1606.50 ± 2.12^b^
		6	2247.50 ± 3.54^e^	102.50 ± 0.71^f^	57.87 ± 0.19^e^	1230.50 ± 2.12^a^	2668.50 ± 2.12^e^	1017.00 ± 1.41^j^	1241.50 ± 2.12^o^
		8	2488.50 ± 3.54^c^	62.50 ± 0.71^l^	50.03 ± 0.04^j^	1233.50 ± 2.12a	2668.50 ± 2.12^e^	1255.00 ± 1.41^c^	1359.50 ± 2.12^m^
		10	2770.50 ± 3.54^a^	62.25 ± 0.35^l^	50.43 ± 0.10^j^	1125.50 ± 2.12^d^	2829.50 ± 2.12^a^	1645.00 ± 1.41^a^	1177.50 ± 2.12^p^
Oxygen	22	0	1898.50 ± 3.54^o^	62.25 ± 0.35^l^	67.93 ± 0.10^a^	1017.50 ± 2.12^l^	2118.50 ± 2.12^q^	1548.00 ± 1.41^b^	949.50 ± 2.12^q^
		2	2045.50 ± 3.54^k^	110.50 ± 0.35^e^	59.57 ± 0.33^d^	1015.50 ± 2.12^l^	2266.50 ± 2.12^p^	885.00 ± 1.41^o^	1360.50 ± 2.12^m^
		4	2101.50 ± 3.54^i^	136.50 ± 0.71^c^	65.20 ± 0.28^c^	1069.50 ± 2.12^h^	2372.50 ± 2.12^o^	883.00 ± 1.41^o^	1451.50 ± 2.12^k^
		6	2159.50 ± 3.54^f^	112.50 ± 0.71^d^	59.90 ± 0.14^d^	1051.50 ± 2.12^j^	2461.50 ± 2.12^n^	987.00 ± 1.41^l^	1518.50 ± 2.12^g^
		8	2325.50 ± 3.54^d^	86.50 ± 0.71^h^	54.31 ± 0.28^g^	1076.50 ± 2.12^g^	2537.50 ± 2.12^j^	1021.00 ± 1.41^i^	1565.50 ± 2.12^e^
		10	2721.50 ± 3.54^b^	146.25 ± 0.35^b^	67.27 ± 0.33^ab^	1094.50 ± 2.12^f^	2565.50 ± 2.12^g^	1024.00 ± 1.41^h^	1586.50 ± 2.12^d^
	25	0	1898.50 ± 3.54^o^	62.25 ± 0.35^l^	67.93 ± 0.10^a^	1017.50 ± 2.12^l^	2118.50 ± 2.12^q^	1548.00 ± 1.41^b^	949.50 ± 2.12^q^
		2	2045.50 ± 3.54^k^	60.50 ± 0.71^m^	50.42 ± 0.12^j^	1038.50 ± 2.12^k^	2456.50 ± 2.12^n^	1007.00 ± 1.41^k^	1422.50 ± 2.12^l^
		4	2101.50 ± 3.54^i^	74.50 ± 0.71^j^	51.78 ± 0.31^i^	1060.50 ± 2.12^i^	2542.50 ± 2.12^i^	1041.00 ± 1.41^g^	1486.50 ± 2.12^i^
		6	2159.50 ± 3.54^f^	146.50 ± 0.71^b^	67.35 ± 0.22^ab^	1077.50 ± 2.12^g^	2550.50 ± 2.12^h^	1082.00 ± 1.41^e^	1477.50 ± 2.12j
		8	2325.50 ± 3.54^d^	110.50 ± 0.71^e^	59.65 ± 0.35^d^	1110.50 ± 2.12^e^	2679.50 ± 2.12^d^	1215.00 ± 1.41^d^	1723.50 ± 2.12^a^
		10	2721.50 ± 3.54^b^	148.50 ± 0.71^a^	67.72 ± 0.40^ab^	1173.50 ± 2.12^b^	2711.50 ± 2.12^b^	1548.00 ± 1.41^b^	1594.50 ± 2.12^c^

The pasting temperature refers to the temperature at which the amylose and amylopectin chains emerge from the granules and the viscosity begins to increase. Table 4 indicates that oxygen‐treated flour samples have a higher pasting temperature. The increase in voltage and time caused a significant increase in the pasting temperature of the samples. It is possible that the placement of functional groups and acidic compounds produced on the surface of the granules initially prevented the release of amylose and amylopectin filaments, and this factor increased the pasting temperature. Wu et al. (2018) investigated the effect of plasma on the rheological properties of banana starch samples. The results of this study showed that with increasing the voltage from 0 to 50, the pasting temperature of starch increased from 74.4 to 92.1°C. The high pasting temperature is as a result of higher crystallinity (Yuan et al., 2007) and higher resistance to water uptake (Seetharaman et al., 2001).

Air‐treated samples have a higher hold viscosity. Also, the increase in voltage caused a significant increase and increase in treatment time caused reduction in the hold viscosity of the samples.

Final viscosity refers to the viscosity of the sample after heating (up to 95°C) and cooling (up to 50°C). As can be seen from the final viscosity results in Table 4, air‐treated samples significantly have higher final viscosity. Increase in voltage and treatment time show a significant increase in the final viscosity of samples. Oxidative reactions on carbohydrates, amino acids, and unsaturated fatty acids result in the production of acidic compounds. The acidic environment increases the bonds between H+ and the alkali ions and starch granules and ultimately the viscosity. Thirumdas, Deshmukh, et al. (2016) and Thirumdas, Saragapani, et al. (2016) also reported the increase in final viscosity of cold plasma‐treated rice starch due to the formation of hydrogen bonds during the plasma process and starch depolymerization. Increasing of intensity and time of plasma treatment leads to increase in peak viscosity of cold plasma‐treated corn starch and tapioca that has been reported by Banura et al. (2018). According to the results of breakdown viscosity in Table 5, air plasma‐treated samples had higher breakdown viscosity. An increase in voltage and treatment time significantly increased the breakdown viscosity of the samples.

As can be seen from the results of setback viscosity in Table 5, oxygen plasma‐treated samples have higher setback viscosity. The results showed that increasing the voltage and treatment time significantly decreased the setback viscosity of the samples. Sudheesh et al. (2019) investigated the effect of plasma on Kithul starch. The results of this study showed that the setback viscosity increased significantly due to the formation of more hydrogen bonds. Wu et al. (2018) investigated the effect of plasma on the rheological properties of banana starch samples. The results of this study showed that with increasing voltage from 0 to 50, the setback viscosity of starch decreased from 51.7 to 21.5. Setback viscosity refers to the degree of reversibility of starch amylose bonds (Charles et al., 2004). Starch with a higher setback viscosity is usually more prone to retrograde. Higher intensity of cold plasma reduces the degree of retrogradation and improves the stability of the dough in cooling (Wu et al., 2018). Sui et al. (2016) investigated the effect of the ozone process on the properties of wheat flour. The results of this study showed that with increasing the time of the ozone process, peak viscosity, drop viscosity, final viscosity, and setback viscosity increased significantly. The reason was reported to be oxidation and production of acidic compounds during it. Misra et al. (2015) also mentioned the effective improvement of mixing time and dough strength after cold plasma treatment mainly attributed to oxidation of protein sulfhydryl groups and subsequent disulfide bond formation between cysteine moieties. Pal et al. (2016) investigated the effect of plasma on the physicochemical properties of rice flour. The results of this study showed that with increasing voltage and time, peak viscosity, breakdown viscosity, final viscosity, and setback viscosity increased significantly. Bahrami et al. (2016) stated that plasma increases the rate of lipid oxidation but the changes did not have a significant effect on RVA parameters and functional properties of wheat flour (Table 5).

## 4 CONCLUSION

Nonthermal plasma treatment improved the properties of sunn pest‐damaged wheat flour, such as color parameters, water absorption capacity, water solubility, oil absorption capacity, swelling strength, pasting time and pasting temperature, and peak, final, and breakdown viscosity of plasma‐treated samples. Setback viscosity was decreased in plasma treatment which reduces retrograde and improves dough cooling stability. Also, the plasma treatment reduced the particle size and the particles of the same size were placed next to each other. Among the studied parameters, the effect of time and type of gas was significantly higher (*p* < .05). Further studies focused on isolated gluten proteins are required to determine the effects of NTP species on its functional properties.

## CONFLICT OF INTEREST

All authors have declared that they do not have any conflict of interest for publishing this research.

## ETHICAL APPROVAL

This study does not involve any human or animal testing.

## Data Availability

The datasets generated are available from the corresponding author upon reasonable request.
